# Evaluation of MHC-II peptide binding prediction servers: applications for vaccine research

**DOI:** 10.1186/1471-2105-9-S12-S22

**Published:** 2008-12-12

**Authors:** Hong Huang Lin, Guang Lan Zhang, Songsak Tongchusak, Ellis L Reinherz, Vladimir Brusic

**Affiliations:** 1 Cancer Vaccine Center, Dana-Farber Cancer Institute, Boston, MA, 02215, USA; 2 School of Land, Crop and Food Sciences, University of Queensland, Brisbane, Australia

## Abstract

**Background:**

Initiation and regulation of immune responses in humans involves recognition of peptides presented by human leukocyte antigen class II (HLA-II) molecules. These peptides (HLA-II T-cell epitopes) are increasingly important as research targets for the development of vaccines and immunotherapies. HLA-II peptide binding studies involve multiple overlapping peptides spanning individual antigens, as well as complete viral proteomes. Antigen variation in pathogens and tumor antigens, and extensive polymorphism of HLA molecules increase the number of targets for screening studies. Experimental screening methods are expensive and time consuming and reagents are not readily available for many of the HLA class II molecules. Computational prediction methods complement experimental studies, minimize the number of validation experiments, and significantly speed up the epitope mapping process. We collected test data from four independent studies that involved 721 peptide binding assays. Full overlapping studies of four antigens identified binding affinity of 103 peptides to seven common HLA-DR molecules (DRB1*0101, 0301, 0401, 0701, 1101, 1301, and 1501). We used these data to analyze performance of 21 HLA-II binding prediction servers accessible through the WWW.

**Results:**

Because not all servers have predictors for all tested HLA-II molecules, we assessed a total of 113 predictors. The length of test peptides ranged from 15 to 19 amino acids. We tried three prediction strategies – the best 9-mer within the longer peptide, the average of best three 9-mer predictions, and the average of all 9-mer predictions within the longer peptide. The best strategy was the identification of a single best 9-mer within the longer peptide. Overall, measured by the receiver operating characteristic method (A_ROC_), 17 predictors showed good (A_ROC _> 0.8), 41 showed marginal (A_ROC _> 0.7), and 55 showed poor performance (A_ROC _< 0.7). Good performance predictors included HLA-DRB1*0101 (seven), 1101 (six), 0401 (three), and 0701 (one). The best individual predictor was NETMHCIIPAN, closely followed by PROPRED, IEDB (Consensus), and MULTIPRED (SVM). None of the individual predictors was shown to be suitable for prediction of promiscuous peptides. Current predictive capabilities allow prediction of only 50% of actual T-cell epitopes using practical thresholds.

**Conclusion:**

The available HLA-II servers do not match prediction capabilities of HLA-I predictors. Currently available HLA-II prediction servers offer only a limited prediction accuracy and the development of improved predictors is needed for large-scale studies, such as proteome-wide epitope mapping. The requirements for accuracy of HLA-II binding predictions are stringent because of the substantial effect of false positives.

## Introduction

Vaccines are the most effective means for fighting against infectious diseases [1]. They are emerging as promising therapies for cancer [2], allergy [3], and autoimmunity [4]. The goal of vaccination is to induce immunity against pathogens and cancer cells by stimulating antigen-specific cytotoxic T lymphocytes (CTLs) or B cells. CTLs recognize peptide antigens presented by major histocompatibility complex class I (MHC-I) molecules on infected cells or cancer cells and kill them. B cells produce antibodies that specifically recognize pathogen- or cancer related molecules. Both these processes are initiated and regulated by T-helper (Th) cells that recognize antigenic peptides presented by MHC class II (MHC-II) molecules. MHC-II molecules present antigenic peptides internalized by professional antigen presenting cells, such as macrophages, dendritic cells, or T lymphocytes. A vaccine must at minimum contain two antigenic epitopes: one to induce specific B-cell or CTL responses and another to induce specific Th cells that regulate (initiate, enhance, or suppress) immune responses [5]. Peptides presented by MHC-I molecules are mainly intracellular and those presented by MHC-II molecules originate mainly from or extracellular proteins. A distinct characteristic of MHC molecules of either class is a groove that binds peptides in a highly promiscuous manner.

The peptide-binding groove of a MHC molecule consists of a β-sheet and two α-helices. A peptide binds through a network of hydrogen bonds between its backbone and the binding groove, and through interactions between the peptide side chains and pockets inside the binding groove [6,7]. Most MHC-I binding peptides are 8–11 amino acids long [8]. MHC-II molecules bind nested sets of peptides most of which are 14–18 amino acids long [9], but some can extend beyond 30 amino acids. MHC-I molecules accommodate the whole length of the binding peptide inside their grooves that are closed [6]. Binding grooves of a MHC-II molecules have open ends; they accommodate the 9-mer binding core of the peptides inside while peptide termini protrude outside of the grooves [7].

The ability of the immune system to respond to a particular antigen differs between individuals because they display different patterns of MHC genes. Human MHC molecules are known as human leukocyte antigens. Each human individual expresses up to six HLA-I molecules and up to a dozen HLA-II molecules. HLA genes show extensive polymorphism. As of August 2008, more than 3000 HLA alleles have been identified and sequenced including 2215 HLA-I and 986 HLA-II sequences [10]. The diversity of HLA molecules increases the probability that any foreign antigen will contain HLA-binding peptides suitable as vaccine targets. The amino acids within the binding groove determine the specificity of peptide binding to a given HLA molecule. Across multiple HLA molecules, the polymorphic residues that form the binding groove determine the repertoire of binding peptides to a particular HLA molecule. Tens of thousands of allele-specific and promiscuous MHC binders and T-cell epitopes have been identified in humans and mice while smaller numbers have been identified in other model animals, such as monkeys and rats [11,12].

Identification of HLA binding molecules is, therefore, important for both understanding the basing molecular function of the immune system and for vaccine development. However, systematic T-cell epitope mapping is costly and time-consuming because it involves synthesis and testing of overlapping peptides spanning the full length of target antigens. For short peptides such as tumor antigen surviving (BIRC5), that is 142 amino acids long, full overlapping studies of both HLA-I and -II binders were performed for several HLA molecules [13,14]. However systematic studies are prohibitively expensive for studies of long antigens, such as autoantigen thyroglobulin (2768 amino acids long), where computational predictions were used to preselect suitable targets followed by experimental validation [15,16]. This problem is particularly pronounced in the studies of whole pathogen proteomes, even in small viruses, such as influenza [17], or dengue [18].

Computational prediction of peptide binding to MHC molecules has been a topic of vigorous research and development activity [19-22]. Computational methods for prediction of HLA-I binding have reached a high level of sophistication and accuracy and represent significant research resources [23]. Computational predictions of HLA-II binding were useful in the study of infectious disease [24,25], cancer [26,27], and autoimmunity [15,16]. However, recent reports have indicated that computational predictions of HLA-II binding are of much lower accuracy than for their HLA-I counterparts [28,29], and even that these predictions may cause more confusion than conclusion [30]. The methods used for assessment of predictors of HLA-II binding have suffered from inadequately defined test sets and testing strategies. Several critical issues need to be addressed to rectify these failings.

• Only a small fraction of peptides in a given pathogen or tumor-specific proteome are able to bind to a specific MHC molecule [31]. Tens of thousands of protein variants have been characterized in viruses [17,18]. Several hundred of tumor-related antigens and their variants have been reported [32,33]. The extensive variability of target antigens significantly increases the number of testable targets, making each individual binding peptide a representative of a large family of individual peptide groups or families [34].

• The comparison studies performed to date have been based on assessing predictive performance using pre-defined sets of peptides, rather than well-defined standardized full-overlapping studies of complete antigens. This introduces biases and the reported performances are likely to be overestimates.

• HLA-II peptide binding is mediated through 9-mer binding core, but longer peptides are used for experimental measurement of binding. Hereby we predict one element (the 9-mer binding cores) and experimentally test with another element (15-mer, or longer peptides). This makes the improvement of false positive rate an important issue in prediction of HLA-II binding and it requires sophisticated statistical and machine learning approaches (see [28,29,34]).

• Both ends of the peptide binding grooves in HLA-II molecules are open, allowing the peptides to be more variable in length (typically 14–18 amino acids) and flanking residues are known to selectively affect binding [9]. This effect is not considered in most of the HLA-II prediction methods.

• Some longer peptides bind MHC-II through multiple overlapping 9-mer registers [34,35] adding further complexity to the selection of actual binding cores. The simpler question of identification of the location of 9-mer binding is extended to identification of multiple binding cores and their locations within the same peptide.

• Experimental measurements of HLA-II binding shows variation depending on the conditions of the experiment, even for the control peptides.

• Sufficient quantities of HLA-II binding data are available only for some HLA-DR molecules while, notwithstanding notable exceptions [35], HLA-DQ and -DP molecules have been understudied.

• Presentation of HLA-II binding peptides depends on antigen processing steps including editing by HLA-DM and other accessory molecules. DM editing affects the density and preference for particular peptide species [36]. These effects have not yet been included in the prediction approaches.

HLA-II binding predictions are thus more complex than HLA-I predictions [23,37,38]. Various prediction algorithms have been developed to facilitate the identification of HLA-II binding peptides within protein antigens. They made computational pre-screening of antigens for HLA-II epitopes a standard approach in epitope-mapping studies; more than twenty prediction servers have been developed to facilitate the identification of MHC-II binding peptides. The performance of six prediction methods has been compared in each of the three recent studies [28-30]. The overall conclusions of these studies were similar, indicating a relatively low prediction accuracy of HLA-II binding predictors. Large quantities of HLA-DR binding peptides with precise measurements have recently become available [28,29], yet contemporary methods have shown little, if any, improvement when compared to the older TEPITOPE method.

This study extends the assessment of predictive power to include a much larger number of servers that predict HLA-II binding. This study was limited to seven common HLA-DR molecules that have sufficient amount and quality of peptide binding data. We compiled and established standardized test data sets that are more representative of the experimental reality, and defined a uniform scaling scheme to use data from different studies. Finally we assessed the practical applicability of HLA-II binding predictions to identification of HLA-II T-cell epitopes. Our study identified several key issues that need to be addressed for the development of improved prediction systems of HLA-II binding.

## Results

### Classification

While not all the servers were designed specifically for peptide binding predictions, all of them have implemented modules for this step. Some servers also have advanced options, for example, MHCPred enables users to specify anchor positions. For this analysis we used the simplest prediction method available at each server. The numbers of the servers for individual HLA-DR alleles we studied were: HLA-DRB1*0101 – 19, HLA-DRB1*0301 – 15, HLA-DRB1*0401 – 20, HLA-DRB1*0701 – 16, HLA-DRB1*1101 – 17, HLA-DRB1*1301 – 9, and HLA-DRB1*1501 – 17.

**Figure 1:**
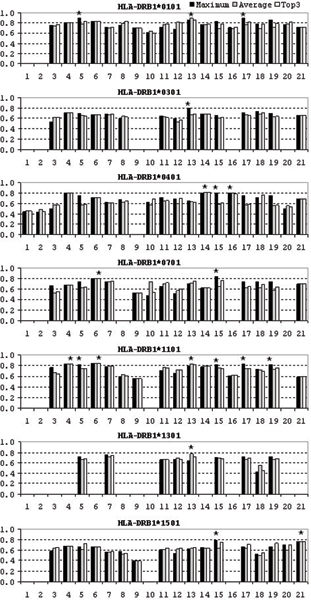
A_ROC _values of predictions by the 21 servers using the combined test set (103 peptides from the four antigens) based on the three mapping methods: black bars for maximum 9-mer scores, grey bars for average scores of all overlapping 9-mers, and white bars for the average of the top three 9-mer scores. Vertical axis shows the A_ROC _values while horizontal axis shows individual servers, as designated in Table 2. Best performing predictors for each allele are marked by asterisks.

In total 113 individual predictors were tested of which 17 showed good, 41 marginal, and 55 poor performance using the single maximum 9-mer prediction scheme. 8 showed good, 30 marginal, and 75 poor performance using the average prediction for all 9-mers within the test peptide. Using the average of best of three 9-mer predictions, 12 servers showed good, 37 marginal, and 64 poor, performance. The A_ROC _values of these predictions are shown in Figure 1. An important finding from this analysis is that overall, for the best prediction scheme (a single best 9-mer), half of the prediction servers are not predictive while only 15% of the servers show acceptable performance. Other prediction schemes show even lower predictive performance.

Comparing the prediction performance across HLA-DR alleles, the best predictors are for HLA-DRB1*0101, where seven predictors showed good classification accuracy, while six DRB1*1101 predictors, three DRB1*0401 predictors, and only one DRB1*0701 predictor showed good classification accuracy. None of predictors for DRB1*0301, DRB1*1301, and DRB1*1501 showed good classification performance. Important to note, only four HLA-DRB1*0101 predictors have shown performance that approaches the value of A_ROC _= 0.9 while other "good" predictors are close to the lower borderline leaving ample space for the improvement.

The best prediction server across all HLA molecules evaluated in this study is NETMHCIIPAN, closely followed by PROPRED, IEDB_SAT, and MULTI_SVM. The best predictors we recommend for each allele are marked by asterisks in Figure 1.

### Prediction of promiscuous peptides

**Figure 2:**
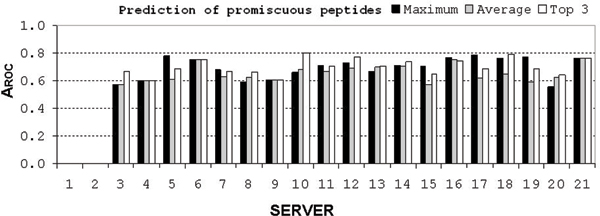
A_ROC _values for prediction of promiscuous peptides. Vertical axis shows the A_ROC _values while horizontal axis shows numbers designating individual servers, as shown in Table 2. The first two servers were excluded from the analysis because they predicted peptide binding to a single DR molecule.

Promiscuous peptides are able to bind to multiple MHC molecules. Therefore they serve as promising targets for vaccine design because they are likely to cover a larger population of patients [39]. We performed analysis of prediction of promiscuous peptides by assigning a score to each peptide, which indicated the number of HLA-DR molecules it binds to. The A_ROC _was then calculated and the results are shown in Figure 2. None of the predictors showed good performance, while MHCPRED, RANKPEP, PROPRED, IEDB_SAT, MULTI_HMM reached A_ROC _values higher than 0.775. DR4_ANN and DR4_SVM predictors were excluded from this analysis since they predict peptide binding to single MHC-II allele (HLA-DRB1*0401). To enable the comparison of predictions that include multiple HLA alleles, we developed a common scaling scheme for seven HLA-DRB1 alleles.  Binding scores used in this scheme range from 0 to 100 and threshold for binding is at 50. The scaled data are accessible at DFRMLI [42].

### Prediction of T-cell epitopes

We also assessed the performance of prediction servers in identification of tumor antigen T-cell epitopes. For each server we predicted the binding affinity of all T-cell epitopes and determined the thresholds at which approximately 80% and 50% of tested T-cell epitopes were predicted as binders. The number of false positives (FPs) at the thresholds was calculated for the four antigens and representative results are shown in Table 1.

**Table 1:** Prediction performance of selected representative servers at two scenarios: a) thresholds that correctly predict ~80% of T-cell epitopes; b) thresholds that correctly predict ~50% of T-cell epitopes.

**Server**	**Threshold**	**TP**	**FN**	**TN**	**FP**	**TP (tumor epitopes)**
**a) 80% prediction**						
IEDB_ARB	100	10	5	56	32	17 (81%)
IEDB_ CON	11	10	5	70	18	17 (81%)
MHCPRED	15	10	5	36	52	17 (81%)
MULTI_ANN	3	15	0	38	50	17 (81%)
MULTI_SVM	6.4	13	2	59	29	17 (81%)
NETMHCII	4000	12	3	51	37	17 (81%)
NETMHCIIPAN	440	12	3	56	32	17 (81%)
PROPRED	-1.5	14	1	54	34	17 (81%)
RANKPEP	0.85	12	3	41	47	17 (81%)

**b) 50% prediction**						
IEDB_ARB	3	7	8	80	8	10 (48%)
IEDB_ CON	4	7	8	86	2	10 (48%)
MHCPRED	3	4	11	76	12	10 (48%)
MULTI_ANN	5	5	10	66	22	11 (52%)
MULTI_SVM	7.1	6	9	82	6	10 (48%)
NETMHCII	400	7	8	78	10	10 (48%)
NETMHCIIPAN	60	7	8	87	1	10 (48%)
PROPRED	0	10	5	81	7	11 (52%)
RANKPEP	7	10	5	69	19	11 (52%)

To identify 80% of T cell epitopes, the threshold for each predictor had to be set low, which resulted in a large number of false positives. This problem was pronounced for predictors such as MHCPRED, MULTI_ANN and RANKPEP, since the number of false positives even exceeded that of true negatives. At this threshold, IEDB (consensus), MULTPRED (SVM), and PROPRED showed the best performance. On the other hand, the thresholds for predicting ~50% of known T cell epitopes were much more stringent, significantly lowering the rate of false positives relative to the 80% threshold. At this threshold, NetMHCIIpan, IEDB (consensus), and PROPRED showed the best performance

## Conclusion and discussion

In this study we evaluated the performance of 21 prediction servers for HLA-II binding peptides. Seven DRB1*0101 predictors, six DRB1*1101 predictors, three DRB1*0401 predictors, and one DRB1*0701 predictor showed good performance in identification of binders and non-binders.  None of predictors for DRB1*0301, DRB1*1301, and DRB1*1501 performed well, indicating that much room for improvement still exists for MHC-II prediction.

The results suggest that some of current predictors are useful for pre-screening Th epitopes, although a relatively large number of false positives (at lower thresholds) and false negatives (at higher thresholds) would also be produced. Predictions using lower thresholds are useful for screening true negatives, while predictions at higher thresholds help cheaply identify a subset of the T-cell epitopes. Unlike MHC-I predictions, we have no evidence that nonlinear methods would perform better than linear methods. One possible reason may be due to the fact that nonlinear methods, such as ANN or SVM, generally require relatively larger amount of data for model development than linear methods. However, the amount of high-quality binding data for MHC-II binding is still far from sufficient, which limits the capability of nonlinear methods to recognize characteristics underlying MHC-peptide interaction

In summary, the prediction accuracy of HLA-II binding peptides is inferior to that of HLA-I binding peptides. Several factors appear to account for this disparity. Insufficient or low-quality training data has been the problem for developers of prediction methods for HLA-II binding peptides. Another problem with HLA-II predictions is the difficulty in identifying 9-mer binding cores within longer peptides used for training as well as lack of consideration of the influence of flanking residues. Amino acids flanking the binding core, contribute to MHC-peptide interactions and also antigen processing preferences [34,40]. Another reason of poor performance for MHC-II prediction is that the binding groove of HLA-II molecules is relatively permissive for peptide binding, which limits the stringency of specific binding motifs. We propose that with new large datasets available [29,37,41,42] new methods that implement knowledge-based strategies and computational search techniques need to be developed. Examples showing various approaches that can improve HLA-binding prediction systems include the use of advanced search algorithms [28,29,43], advanced statistical and machine learning approaches [44-47], combination approaches [28,38,48,49], novel scoring functions [50], and improved use of structural predictions [51,52], or application of knowledge-based approaches [53-57]. Future HLA-DR prediction developments studies should, at minimum, use standardized data sets, provide improved definition of binding cores, minimize number of false positives, and consider the effects of flanking residues.

Results of this study will help researchers to determine the most appropriate servers for pre-screening of HLA class II binding peptides.  In addition, this study has defined basic criteria for slection of predication thresholds for selection of peptides that are most likely to be potential HLA-II epitopes. On the other hand, it provides guidelines for testing and test data to server developers. This knowledge, together with standardized test data sets should empower them to produce better solutions and improve prediction performance. Normalization and standardization methods that we introduced in this study enable annotation and integration of heterogeneous data into a uniform format, which facilitates the development of advanced algorithms. Future advancement in high-throughput measurements of binding affinities is expected to significantly improve the prediction performance of MHC-II binding peptides.

## Materials and methods

We evaluated 21 servers for prediction of HLA class II binding peptides that have been developed by 12 groups (Table 2). These servers were accessible over the Internet as of July 2008. Predictive algorithms used in these servers include: binding matrices, partial least square function, artificial neural networks (ANN), hidden Markov models (HMM), and support vector machines (SVM). Our study involved five consecutive steps: a) Construct test data sets by collecting independent experimental data; b) Retrieve prediction results from the 21 servers; c) Assess the classification accuracy (binders *vs*. non-binders); d) Assess the prediction accuracy of promiscuous binding affinities; e) Assess the performance for predicting T cell epitopes.

**Table 2:** List of prediction servers of HLA class II binding peptides, their URLs (as of December 2007), and name abbreviations.

**ID**	**Servers**	**Abbreviation**	**URLs**	**Prediction algorithm**	**Reference**
1	HLA-DR4Pred (ANN)	DR4_ANN	[63]	ANN	[64]
2	HLA-DR4Pred (SVM)	DR4_SVM	[63]	SVM	[64]
3	IEDB (ARB)	IEDB_II	[65]	Matrix	[66]
4	IEDB (SMM)	IEDB_SMM	[65]	Matrix	[41]
5	IEDB (Saturniolo)	IEDB_SAT	[65]	Matrix	[67]
6	IEDB (Consensus)	IEDB_CON	[65]	Matrix	-
7	MHC Binder Prediction	MHC_BP	[68]	Matrix	-
8	MHC2Pred	MHC2Pred	[69]	SVM	-
9	MHC-BPS	MHC_BPS	[70]	SVM	[71]
10	MHCPred	MHCPRED	[72]	Partial least square	[73]
11	Multipred1 (ANN)	MULTI_ANN	[74]	ANN	[39]
12	Multipred1 (HMM)	MULTI_HMM	[74]	HMM	[39]
13	Multipred1 (SVM)	MULTI_SVM	[74]	SVM	[75]
14	NetMHCII	NETMHCII	[76]	Matrix	[41]
15	NetMHCIIpan	NETMHCIIPAN	[77]	ANN	[29]
16	PeptideCheck (Matrix)	PEPC_M	[78]	Matrix	[79]
17	ProPred	PROPRED	[80]	Matrix	[81]
18	Rankpep	RANKPEP	[82]	Matrix	[83]
19	SVMHC	SVMHC	[84]	Matrix	[85]
20	SVRMHC	SVRMHC	[86]	SVM	[87]
21	SYFPEITHI	SYFPEITHI	[88]	Matrix	[12]

### Data sets

In this study our test data sets consisted of 103 peptides derived from four protein antigens, including allergens – bee venom phospholipase A2 (API m1) [58] and dog lipocalin (Can f 1) [59], a tumor antigen LAGE-1 [60], and a viral antigen HIV NEF [61]. Although these studies were done by different groups, they were performed using comparable protocols and same control peptides. The lengths of the studied peptides were in the range of 15 to 19 amino acids (Table 3). Binding capability of these peptides to corresponding HLA molecules was measured by the concentration of peptides that prevented binding of 50% of the labeled reference peptides. These studies reported binding data for seven HLA-DR molecules (DRB1*0101, 0301, 0401, 0701, 1101, 1301, and 1501). The test data sets used in this study were extracted from the original references and rescaled to a common scale. The data used in this study are accessible at the Dana-Farber Machine Learning Repository for Immunology (DFRMLI) [42].

**Table 3:** Summary of the four testing protein antigens

**Antigen**	**Number of peptides**	**Length of peptides**
Phospholipase A2	30	18
LAGE-1	17	16–19
Lipocalin	25	16
HIV NEF	31	15–16

### Predictions and comparisons

Each protein sequence was submitted to the prediction servers and the results were recorded. Most servers predict binding affinities of 9-mer peptides while the experiments were conducted on longer peptides ranging from 15 aa to 19 aa. Three mapping methods were explored to map the 9-mer predictions to experimental results. First, the highest prediction score of the overlapping 9-mer peptides spanning the length of a longer peptide was used as the predicted binding of the longer peptide. Second, the average score of the overlapping 9-mers was used as the predicted binding. Finally, the average of the top three predicted 9-mer scores of the overlapping peptides was used as the prediction score.

Prediction accuracy is measured in terms of the area under the receiver operating characteristic curve (A_ROC_) [62]. The ROC curve is a plot of the true positive rate TP/(TP+FN) on the vertical axis *vs. *false positive rate FP/(TN+FP) on the horizontal axis for the full range of the decision thresholds. The values A_ROC _≥ 0.9 indicate excellent, 0.9 > A_ROC _≥ 0.8 good, 0.8 > A_ROC _≥ 0.7 marginal and 0.7 > A_ROC _poor predictions [62].

In this study we defined promiscuous peptides as those peptides from the test set that bound four or more of the seven studied alleles. Binding was defined as half maximal inhibitory concentration (IC_50_) lower than 100 nM (for DRB1*0101, 0401, 0701, and 1501), or lower than 1000 nM (for DRB1*0301, 1101, and 1301).

### Scaling

To enable visual inspection for comparisons of predictions, both experimental measurements and predictions have been scaled to a common scale from 0 to 100 by linear transformation of the value ranges using the formula for each individual peptide:

$$y_{i}^{S}=\frac{y_{i}-y_{\min}}{y_{\max}-y_{\min}}\times 100$$

where $y_{i}^{S}$ is the scaled score, *y*_*min *_is the minimum and *y*_*max *_is the maximum score. The experimental binding affinity was corrected for variation in binding affinity of control peptides between different experiments then scaled. All values are accessible at DFRMLI site.

## Competing interests

The authors declare that they have no competing interests. Previously HHL co-developed MHC_BPS, GLZ and VB co-developed MULTIPRED, and ELR co-developed Rankpep.

## Authors' contributions

VB and ELR designed the study, HHL performed the analysis, GLZ and ST collected and prepared data. HHL and VB drafted the article and all authors participated in manuscript.
